# Risk factors associated with medium- to long-term outcome and health-related quality of life of patients with conservatively treated rib fractures

**DOI:** 10.1038/s41598-025-92622-4

**Published:** 2025-03-17

**Authors:** Benedikt Heyart, Cathleen Kmezik, Tobias Schöbel, Sebastian Krämer, Christian Kleber, Ulrich J. Spiegl

**Affiliations:** 1 Department of Orthopaedics, Trauma Surgery and Plastic Surgery, University Hospital Leipzig, 04103 Leipzig, Germany; 2 Division of Industrial Biotechnology, Department of Biology and Biological Engineering, Chalmers University of Technology, Gothenburg, 41296 Sweden; 3 Department of Visceral, Transplant, Thoracic and Vascular Surgery, Division of Thoracic Surgery, University Hospital Leipzig, 04103 Leipzig, Germany

**Keywords:** Rib fracture, Conservative treatment, Health-related quality of life, Medium- to long-term outcome, Risk factors, Trauma, Pain, Ageing

## Abstract

Patients with rib fractures often suffer from prolonged pain and dyspnea. The purpose of this study was to evaluate the mid- and long-term outcomes and major predisposing risk factors for clinical limitations such as pain or reduced lung capacity in patients with conservatively managed rib fractures to provide a basis for optimizing current therapy. Patients who underwent conservative management of rib fractures between 2014 and 2018 at a level I trauma center were retrospectively reviewed. Inclusion criteria were Injury Severity Score (ISS) ≤ 16 points and a minimum follow-up of 3 years. Outcome parameters were the SF-36 physical and mental component summary score (PCS and MCS, respectively) as well as current pain and respiratory problems. Risk factors evaluated included age, body mass index (BMI), in-hospital days, number of rib fractures, fracture dislocation and serial rib fracture. PCS was comparable to the normal population. The correlation between age and PCS was significant, *p* = .002. BMI correlated significantly with PCS, *p* < .001, current pain, *p* = .034 and respiratory problems, *p* = .029. No significant correlations were observed for the number of rib fractures and in-hospital days. Fracture dislocation and serial rib fracture showed no effect on PCS, *p* = .134 and *p* = .914, respectively, and current pain, *p* = .916 and *p* = .357, respectively. In the medium- to long-term, conservative treatment of simple rib fractures or serial rib fractures showed good results, but was negatively affected by a high patient age or BMI.

## Introduction

Thoracic injuries account for approximately 10% of all trauma-related hospital admissions worldwide^1,2^. In Europe 90% of thoracic injuries are blunt chest trauma^3^, of which 10–39% show a prevalence of rib fractures^2^. As most statistics do not include outpatients, the prevalence, particularly of isolated rib fractures, is likely to be higher. In addition, chest X-rays performed in patients with minor chest trauma have limited sensitivity in detecting rib fractures^4^, increasing the chance that rib fractures remain undetected.

Patients with rib fractures present mainly with pain and dyspnoea. Consolidation of rib fractures usually takes 6 to 8 weeks, after which complete remission of symptoms is expected^5^. However, several studies have shown that patients can suffer much longer from pain and dyspnea, which negatively affects their quality of life^5–7^. Therefore, alternative treatment options to classical conservative management have been established. Studies of outcomes in trauma patients who have undergone surgical stabilisation of rib fractures have shown reasonable results, but have mostly included polytrauma patients^8,9^. Studies assessing the health-related quality of life in patients with simple rib fractures and serial rib fractures without relevant extrathoracic injuries remain underrepresented.

The aim of this study was to evaluate the medium- and long-term outcome of patients who had experienced rib fractures and to thereby gain insights into the efficiency of the current conservative therapy. In addition to the severity and extent of the fractures, the influence of other patient-specific variables that affect quality of life, such as age and BMI, was examined. The role of these variables as possible risk factors for clinical limitations was investigated, as well as whether the variables could serve as suitable criteria for adapting the treatment concept.

## Methods

### Ethical consideration

The study was approved by the Institutional Ethics Committee of University Hospital Leipzig (internal reference number: No. 420/20-ek).

### Patients

Patients in the study were treated for rib fractures between January 2014 and December 2018 at a Level I trauma center. Inclusion criteria for this study were age greater than 18 years, Injury Severity Score (ISS) less than or equal to 16, a minimum follow-up of 3 years and a conservative therapy of the fractures. Patients with an AIS of 4 and therefore an ISS of 16 were only included if they suffered a thoracic monotrauma, so that the definition of polytrauma did not apply. The conservative therapy for rib fractures consisted of initial imaging diagnostics, pain treatment according to the WHO analgesic ladder and physiotherapeutic respiratory therapy, e.g. periodically usage of breathing devices or CPAP therapy. Depending on the severity of the injury and symptoms, patients were admitted to the intensive care unit (ICU) if necessary. Additional thoracic injuries in some patients included pulmonary contusion, pneumothorax, hemothorax, chest contusion, sternal fracture, scapula fracture, clavicle fracture, and pneumomediastinum. Extrathoracic injuries included traumatic brain injury, fractures, abdominal organ contusion, spinal deformity, soft tissue injury, and contusion. The duration of pain therapy was adjusted according to the individual needs of the patients. Exclusion criteria for this study were flail chest, pregnancy, dementia, mental illness or physical disability, cancer, spontaneous fractures and suicidal tendencies.

### Data collection

Patient information was extracted from the internal database. Study patients also provided information on health-related quality of life, current pain and respiratory function using the German version of the 36-item Short-Form Health Survey (SF-36) and a visual analog scale (VAS).

### Studied variables

The physical health-related quality of life (PCS), which was calculated from the SF-36 as well as the mental health-related quality of life (MCS), was used as main outcome parameter. PCS and MCS scores of 50 are defined as the average quality of life of the general population. Values above, reflect a better quality of life, values below, a lower one. The physical current pain and current respiratory function were measured on a ten-point numeric rating scale. Values of 1 corresponded to no pain or shortness of breath, respectively, and values of 10 corresponded to maximum pain or rapidly occurring shortness of breath, respectively. Patient characteristics of interest and potential risk factors were current age, age at hospital admission, BMI, number of days in hospital, number of rib fractures, fracture dislocation, and presence of serial rib fractures. Fracture dislocation was defined as a displacement of one shaft width.

Of the 105 patients, 10 patients were excluded from PCS calculations due to insufficient responses to the SF-36 questionnaire. 20 patients were excluded from BMI calculations due to missing BMI data. In addition, fracture dislocation could not be determined in four patients.

### Statistical analysis

Statistical analysis was performed using IBM^®^ SPSS^®^ Statistics 27 (IBM Corporation, Armonk, NY, USA). A *p*-value of less than 0.05 was defined as significant. Metric patient demographics were illustrated as mean (*M*) with standard deviation (*SD*) for bivariate normal distributed data and as median (*Mdn*) with interquartile range (*IQR*) for non-normally distributed data. The Kolmogorov-Smirnov test was used to test for normal distribution. Absolute numbers and percentages were used for categorical data.

The Student’s *t* test was applied to the PCS data for comparison with the 1994 German population. An F-test was used to test for equality of variance. In the case of unequal variances in a sample, a Welch test was performed to generate the t-test statistic. Three subgroups were defined according to the German population of 1994 with at least 20 study patients. These age groups were 51 to 60 years, 61 to 70 years, and over 70 years.

Pearson correlation was performed for the metric patient data age at hospital admission, BMI, days in hospital, number of rib fractures, and the outcome parameters PCS, MCS, current pain, and current respiratory function. Correlation coefficients were calculated by BCa bootstrapping with 2,000 BCa samples to generate 95% confidence intervals (CI). Means and standard deviations were used.

Student’s t-test was used to test the influence of fracture severity on physical outcome. Patient variables were fracture dislocation and the presence of serial rib fractures. Physical outcome parameters were PCS and current pain. Equality of variance (homoscedasticity) was determined by Levene’s test.

## Results

Of the 1,130 patients treated for rib fractures, 319 met the inclusion criteria, and 105 agreed to participate in the study. The mean age of the studied patient group was 59.9 years (*SD* = 15.9) at hospitalization with a mean BMI of 26.7 kg/m^2^ (*SD* = 4.2). Patients had a median of 3 broken ribs (*IQR* = 4), most of which were non-dislocated (88.1%) and nearly half of which were serial rib fractures (47.6%). The median in-hospital stay was 5 days long (*IQR* = 5). Some patients had preexisting lung or cardiovascular disease (17.1%). The median PCS as the main outcome parameter was 47.8 (*IQR* = 17). The median MCS was 52.0 (*IQR* = 15). Current pain and respiratory problems had a median value of 2.0 (*IQR* = 3) and 1.0 (*IQR* = 4), respectively. All patient characteristics are shown in Table 1.

**Table 1:** Patient characteristics.

Variable	*n*	%	M	SD	Mdn	IQR
Patient variables						
Age at hospitalization (a)			59.9	15.9		
Subgroups: Current age (a)						
51–60 years	24	29.6	55.7	2.7		
61–70 years	22	27.2	66.1	3.0		
> 70 years	35	43.2	79.8	5.7		
BMI (kg/m^2^)			26.7	4.2		
Number of fractures					3.0	4
In-hospital days (d)					5.0	5
Preexisting lung or cardiovascular disease						
No	87	82.9				
Yes	18	17.1				
Dislocation						
No	89	88.1				
Yes	12	11.9				
Serial rib fracture						
No	55	52.4				
Yes	50	47.6				
Outcome parameter						
PCS					47.8	17
MCS					52.0	15
Current pain					2.0	3
Current respiratory problems					1.0	4

BMI = Body mass index; PCS = Physical component summary; MCS = Mental component summary. The PCS and MCS values were calculated from the subjects’ responses to the Short Form 36 (SF-36) health status questionnaire and are dimensionless. Scores for “Current pain” and “Current respiratory problems” were queried by visual analog scale (scale range, 1 to 10) and are dimensionless.

**Fig. 1:**
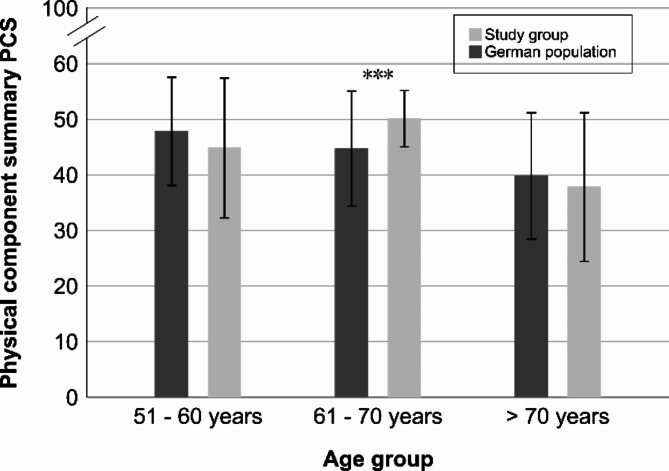
PCS divided by age groups for the studied patient group and the German population. Error bars represent standard errors. *** *p* < .001.

Figure 1 shows the comparison of individuals in the study group and the German population. In the 51–60 year old study group the PCS was 44.9 (*SD* = 12.5) for the study participants and 47.9 (*SD* = 9.7) for the norm sample. The difference was not statistically significant. The mean PCS score for study patients with aged between 61 years and 70 years was 50.2 (*SD* = 5.1), which was significantly higher than the norm sample (*M* = 44.8, *SD* = 10.3); t(31) = 4.50, *p* < .001. Patients over 70 years of age showed a slightly higher PCS score in the norm sample compared to the study group, but the observed difference was not statistically significant. Both scores were slightly below 40 (rib fractures: *M* = 37.9, *SD* = 13.3; German population: *M* = 39.9, *SD* = 11.3).

The correlation results are listed in Table 2. Factors significantly correlated with PCS were age at hospital admission and BMI. Overall, there was a moderate negative correlation between age and PCS, r(71) = − 0.35, *p* = .002. For BMI and PCS, the correlation was also negative with moderate to strong significance, r(71) = − 0.44, *p* < .001. In contrast, neither the number of fractures nor the number of days in hospital correlated with PCS. There were no significant correlations between any of the factors examined in this study and MCS. A significant correlation between a patient-specific characteristic and their current pain and dyspnea was found only in relation to BMI. These correlations were positive and weak to moderate according to Cohen, both for pain, r(78) = 0.24, *p* = .034, and for dyspnea, r(78) = 0.24, *p* = .029.

**Table 2:** Mean, standard deviation, and correlation coefficients for metrically distributed study variables.

Variable		M	SD	1	2	3	4	5	6	7
1.	Age at hospitalization (a)	59.9	15.9	–						
2.	BMI (kg/m^2^)	26.7	4.2	0.13 [-0.10, 0.36]	–					
3.	Number of fractures	3.3	2.3	0.15 [-0.04, 0.32]	0.18 [-0.05, 0.38]	–				
4.	In-hospital days (d)	4.9	3.6	0.24* [0.05, 0.40]	0.04 [-0.11, 0.17]	0.36** [0.12, 0.54]	–			
5.	PCS	44.2	12.1	− 0.35** [-0.53, − 0.15]	− 0.44*** [-0.59, − 0.26]	− 0.08 [-0.30, 0.14]	0.06 [-0.16, 0.27]	–		
6.	MCS	49.8	10.6	0.07 [-0.15, 0.27]	− 0.21 [-0.49, 0.12]	− 0.07 [-0.34, 0.22]	0.11 [-0.09, 0.30]	0.43*** [0.24, 0.62]	–	
7.	Current pain	2.6	2.2	0.12 [-0.12, 0.35]	0.24* [-0.03, 0.44]	0.06 [-0.15, 0.26]	− 0.05 [-0.21, 0.13]	− 0.54*** [-0.07, − 0.34]	− 0.36** [-0.58, − 0.12]	–
8.	Current respiratory problems	2.6	2.4	0.15 [-0.14, 0.40]	0.24* [0.05, 0.43]	0.06 [-0.13, 0.28]	0.13 [-0.05, 0.32]	− 0.56*** [-0.73, 0.35]	− 0.28* [-0.49, − 0.08]	0.56*** [0.33, 0.73]

BMI = Body mass index; PCS = Physical component summary; MCS = Mental component summary. Numbers in square brackets represent the 95% confidence interval of the associated correlation coefficient. Confidence intervals per BCa bootstrapping with 2,000 BCa samples. The PCS and MCS values were calculated from the subjects’ responses to the Short Form 36 (SF-36) health status questionnaire and are dimensionless. * *p* < .05. ** *p* < .01. *** *p* < .001.

**Fig. 2:**
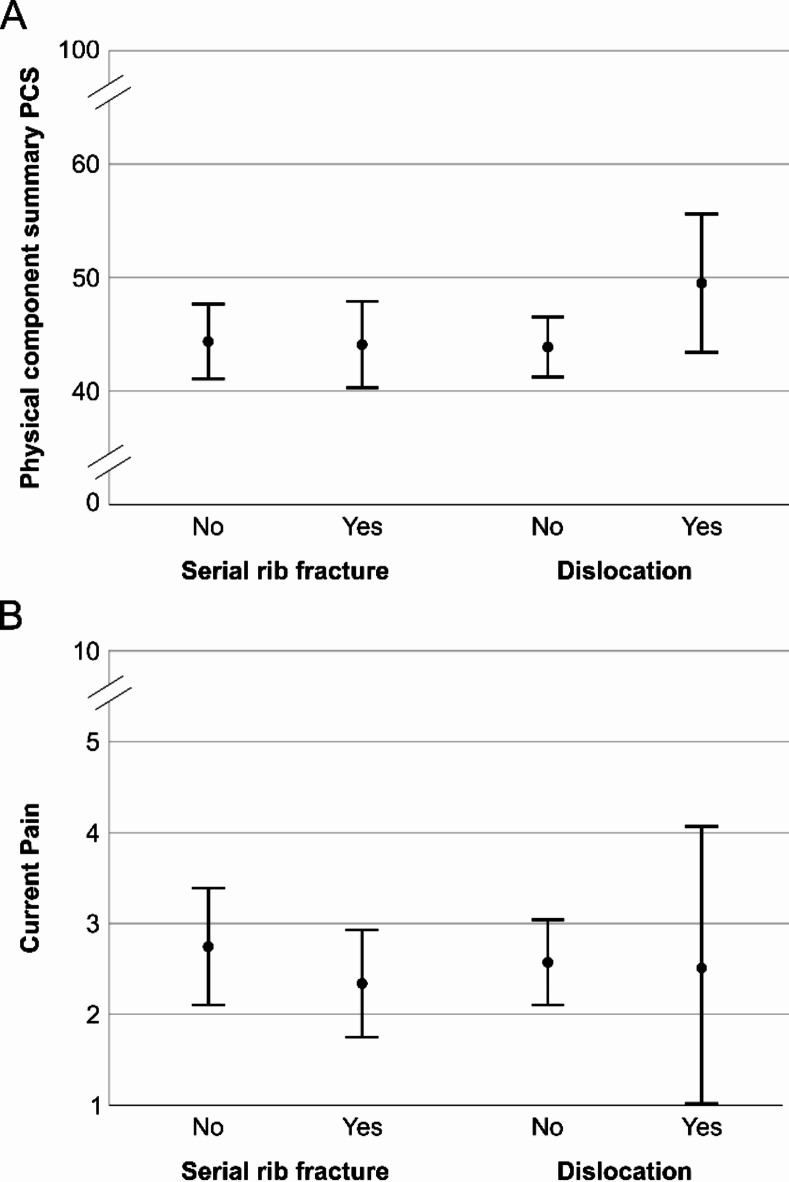
Mean value and standard deviation of PCS (A) and current pain (B) for the serial rib fracture and dislocation subgroups. Error bars represent 95% confidence interval.

Patients with dislocated rib fractures showed higher PCS scores (*M* = 49.5, *SD* = 9.1 vs. *M* = 43.9, *SD* = 11.9) and lower pain (*M* = 2.5, *SD* = 2.5 vs. *M* = 2.6, *SD* = 2.2) compared to non-dislocated fractures, without differing significantly (Fig. 2). PCS scores within the serial rib fracture subgroup were around 44 in both categories. Current pain was not significantly higher in patients without serial rib fractures (*M* = 2.8, *SD* = 2.4 vs. *M* = 2.3, *SD* = 2.1).

Patients with a hemothorax or pneumothorax who underwent treatment via drainage exhibited a non-significantly lower PCS in comparison to those who did not receive such treatment (*n* = 18, *M* = 48.6, *SD* = 8.4 vs. *n* = 12, *M* = 53.1, *SD* = 8.7).

## Discussion

Over the past few decades, the management of rib fractures has undergone some realignments in clinical practice. While conservative methods remain the predominant approach^10^, surgical fixation is receiving increasing attention^11^. According to established medical guidelines and besides individual decisions, the primary indication for surgical stabilization is the presence of flail chest with mechanical respiratory failure^12^). Surgical treatment of this type of fracture has been shown to significantly reduce prolonged pain and pulmonary complications^8,9^. Furthermore, it has been demonstrated that surgical intervention results in enhanced patient quality of life at both one- and six-month follow-ups when compared to the cohort treated conservatively^9^. Several studies have provided evidence that patients with multiple fractures may also benefit from surgical intervention^7,8,13^. However, the majority of fractures, especially simple fractures, are nowadays still treated conservatively through a combination of pain management and respiratory exercises. To the best of our knowledge, this is the first study to examine and evaluate the long-term outcomes, particularly health-related quality of life, of patients with conservatively managed rib fractures without relevant extrathoracic injuries. Additionally, the assessment of key risk factors was an important objective to provide a clinical basis for identifying patients at higher risk of persistent symptoms.

Our study shows that patients with rib fractures without polytrauma are adequately treated with conservative therapy in the long term. Although there is no surgically treated control group, in the medium- to long-term the patients in the study showed a quality of life comparable to that of the normal German population^14^, which supports the efficacy of conservative therapy. Contrary to the other results and the existing literature, the 61–70 age group had the highest quality of life scores, which were even higher than those of the reference group. At the same time, the number of subjects in this age group was the lowest and particularly underrepresented. It is plausible that this cohort contained a significant proportion of people with a high level of physical health, resulting in a statistically favorable quality of life outcome.

Although the main symptoms of pain and respiratory problems were mild, it is not clear whether these mild symptoms were directly caused by the chest trauma or were due to independent risk factors such as preexisting lung and cardiovascular disease. Overall, similar factors were found to influence medium- to long-term PCS, pain, and respiratory problems after rib fracture. No significant correlations were observed between any of the outcome parameters and rib fracture severity. Even fracture associated concomitant injuries that necessitated treatment resulted in no significant PCS change. These findings suggest that the severity of rib fracture in patients without polytrauma may not be the most important factor in determining the optimal management strategy, whether conservative or surgical. On the contrary, the data presented in this study highlight the importance of patient-specific characteristics such as age and BMI as risk factors. Considering these valuable findings, we suggest that a comprehensive assessment including patient-specific factors as identified in this study should be prioritized when choosing between treatment options to promote symptom-free and improved quality of life.

The same influence of age on PCS and MCS has previously been observed in the German norm population^14^ as well as in trauma patients^15^. In patients with isolated rib fractures^16^, an age over 65 years was found to be associated with prolonged pain and respiratory complications after trauma^17,18^. Studies investigating the influence of BMI have only been conducted for the general population. They have demonstrated a negative association with PCS^19,20^, respectively a positive association with increased pain^21,22^ and respiratory problems^23^. In the present study, the number of rib fractures, the occurrence of dislocation, and the presence of a serial rib fractures were examined as parameters of fracture severity, respectively trauma severity. Previous findings already demonstrated that the number of rib fractures exerts no influence on pain intensity in the short-term^6,16^. Other studies examining the quality of life outcome after orthopaedic trauma mostly included a general, non-rib-specific trauma population. However, they have shown that the ISS as a parameter of trauma severity is not a prognostically relevant factor for PCS^15,24,25^, which provides support for our findings. Regarding hospital length of stay, we either could not find relevant literature or found it of limited value for comparison with our study due to differences in study design.

The significance of the results presented here is somewhat limited by the retrospective nature of this study. Confounding variables that may be associated with quality of life (such as occupational status, social support, income, alcohol or smoking status) were excluded from this study. In addition, accidental events or pre-existing conditions that may have occurred in the period after the rib fracture were not recorded. The evaluation of the study population in certain categories was hindered by the presence of missing data in both the questionnaires and the patient-specific data from the medical records. The generalization of the data to all non-polytrauma patients with rib fractures is problematic because the data represent only patients treated at the level I trauma center, which may limit its applicability to other settings. The relatively small sample size of patients available may have diminished the statistical power of this study. In addition, the inclusion of a surgically treated control group could have provided valuable insights, facilitating a more comprehensive assessment of the efficacy of conservative management. Another limitation is the lack of standard follow-up measures for fracture consolidation or other physical outcome parameters that would have allowed a more quantifiable and comparable assessment of pulmonary status. Finally, respiratory complaints may be difficult to distinguish from non-pulmonary causes in a retrospective setting, particularly in the absence of pulmonary function analysis.

Although the above limitations must be taken into account, the results of this study provide valuable insights into the management of rib fractures. In particular, we have shown that conservative management of simple or serial rib fractures leads to good results in the medium- to long-term, but that the treatment outcome may be negatively affected by high patient age or BMI.

Future studies could evaluate short-term management to complement the results presented here. In addition, a comparison of different conservative treatment approaches, with consideration for patient-specific factors such as age and BMI, could provide valuable insights that would facilitate for more optimal patient-specific treatment.

## Conclusion

In the medium-term, most patients achieved satisfactory quality of life and reported low levels pain or respiratory problems following conservative treatment of rib fracture or rib serial fracture without relevant extrathoracic injuries. The fracture severity did not influence the outcome; however, trauma-independent patient characteristics, including age and BMI, were found to be significant risk factors for clinical limitations.

## Data Availability

The datasets generated analyzed during the study are available from the corresponding author on reasonable request.

## Abbreviations

BCa: Bias-corrected and accelerated
BMI: Body mass index
CI: Confidence interval
ICU: Intensive care unit
ISS: Injury severity score
IQR: Interquartile range
MCS: Mental component summary
PCS: Physical component summary
SF-36: 36-Item Short-Form Health Survey
VAS: Visual analogue scale
